# Linking Intensive Care Unit functional scales to the International Classification of Functioning: proposal of a new assessment approach

**DOI:** 10.1186/s12913-023-09787-9

**Published:** 2023-08-16

**Authors:** Juliana S. F. dos Santos, Gabriely A. G. Silva, Nubia M. F. V. Lima, Lucien P. Gualdi, Diego de S. Dantas, Íllia N. D. F. Lima

**Affiliations:** 1 Faculdade de Ciências da Saúde do Trairi, Universidade Federal do Rio Grande do Norte, Santa Cruz, Brasil; 2 Departamento de Fisioterapia, Universidade Federal do Rio Grande do Norte, Campus Universitário, Lagoa Nova, Natal, 59078-970 Brasil; 3 Departamento de Fisioterapia, Universidade Federal de Pernambuco, Recife, Brasil

**Keywords:** International Classification of Functioning, Disability and Health, Intensive Care Unit, Physical therapy

## Abstract

**Background:**

There are several tools to assess functional and physical status in critical ill patients. These tools can guide rehabilitation strategies in Intensive care units (ICU). However, they are not standardized, and this can compromise their applicability. The aim of the study is to identify common contents between International Classification of Functioning, Disability and Health (ICF) and Medical Research Council sum score (MRC-ss), Functional Status Score for the ICU (FSS-ICU), and Physical Function in ICU Test-scored (PFIT-s). As well as to propose a new assessment approach based on the ICF to ICU patients.

**Methods:**

Pilot cross-sectional study. ICU in-patients, both genders, aged between 50 and 75 years were assessed with MRC-ss, FSS-ICU, PFIT-s and the linking rules used were proposed by Cieza et al. The inter-rater agreement for the linking process was performed using the Kappa coefficient.

**Results:**

The ICF categories identified in the tools covered a total of 14 items. Common contents were identified in 13 of the 14 and two were related to body functions, six to body structures and five to activities and participation. The inter-rater agreement was considered substantial for the linking of MRC-ss (k = 0.665) and PFIT-s (k = 0.749) to the ICF, and almost perfect for the FSS-ICU (k = 0.832).

**Conclusions:**

This study synthesizes and categorizes commonly used tools and presents a new proposal based on the ICF to guide future studies. The proposed model combines the ICF with the contents of the most relevant instruments used in critical care.

## Background

Patients admitted to Intensive care units (ICU) are often exposed to bed restriction or immobilism. Extended duration in this condition can lead to ICU acquired weakness (ICU-AW), inflammatory response syndrome (SIRS), multiorgan dysfunction, increased risk of transitory or permanent impairment in physical function or functionality, and development of comorbidities or sequelae that may last for up to five years after hospital discharge [1, 2]. Thus, assessing functional capacity in these patients is crucial because it can guide rehabilitation strategies and reduce length of hospital stay, morbidity, mortality and healthcare costs [3].

Assessment tools are constantly created to objectively measure functionality. Such measurement can be, for example, the ability to maintain and/or recover the performance of basic tasks. These tools also make it possible to quantify the evolution of the functional state and the response to treatments during hospitalization [4–7]. Currently, there are at least thirty-three assessment tools for the ICU scenario. Of these, only 20 have clinometric properties and six were created specifically to assess functionality in critically ill patients [8].

Despite the large number of instruments, their applicability is still controversial. In this context, the possibility of producing standardized and comparable data led international efforts to create the International Classification of Functioning, Disability and Health (ICF). It is a conceptual framework that comprises a biopsychosocial and holistic context and has a common language to describe and classify health and disability [9, 10]. However, due to its huge extension (more than 1400 categories), strategies have been adopted to simplify the ICF use in different contexts and conditions [10]. In addition to the creation of core-sets [11–15] and questionnaires based on the ICF [15], the identification of ICF contents in different instruments has been a widespread alternative in the scientific community since it allows data interpretation within the ICF classification [16–18].

Shortlists of ICF categories and linkage of common instruments used by intensive care professionals to the ICF may encourage its use in these settings. This alternative may save healthcare providers’ time and improve assessment in different hospitalization moments, as well as between different centers. All this facilitates the understanding and the management of functionality in critically ill patients. Furthermore, ICF qualifiers can produce feasible indicators and enable reliable results among different centers [9, 15].

Considering this, the present study aimed to identify common contents between the ICF and the Medical Research Council sum score (MRC-ss), the Functional Status Score for the ICU (FSS-ICU) and the Physical Function in ICU Test-scored (PFIT-s), using the ICF linkage rules proposed by Cieza et al. [16–18]. In addition, critically care patients were assessed and physical function was described using a new proposal based on the ICF.

## Methods

The study was carried out between July and October 2019 at Hospital Regional Dr. Mariano Coelho (Brazil). The research was approved by the Research Ethics Committee of the Federal University of Rio Grande do Norte (CAAE 49235715.3.0000.5568) and conducted in accordance with Resolution 466/12 of the National Health Council and the Declaration of Helsinki of the World Medical Association. All participants signed a consent form.

This is a pilot cross-sectional study with non-probabilistic sampling. In-patients both genders aged between 50 and 75 years, who were able to understand and obey commands, and with controlled blood pressure (i.e., systolic blood pressure between 90mmHg and 180mmHg) were included [19]. Patients with previous history of hospitalizations for more than seven days, with neurological signs or diseases (e.g., stroke, Parkinson’s disease, spinal cord injury or spasticity), with orthopedic disabilities that could interfere in the assessments (e.g., amputations, leg length discrepancy, immobilizations or external fixators), or with cognitive impairments were excluded from the study.

All participants were assessed with the three instruments proposed, with a total average duration of 60 min. Each participant was evaluated between 1 and 7 days after admission to the ICU, when they were hemodynamically stable, able to respond to basic commands and out of mechanical ventilation for more than 48 h.

### Instruments

The MRC-ss was used to assess peripheral muscle strength using manual resistance. This scale presents excellent inter-rater reliability in the ICU environment and is highly correlated with physical function, functionality and hospital length of stay [8, 20]. Muscle contraction strength is scored from 0 to 5 (0 represents no perceived muscle contraction and 5 optimal muscle strength) and it is applied in six muscle groups bilaterally (shoulder abduction, elbow flexion, wrist extension, hip flexion, knee extension and ankle dorsiflexion). The total score ranges from 0 points (complete tetraparesis) to 60 points (normal muscular strength), with scores between 37 and 48 indicating significant weakness and ≤ 36 indicating severe weakness [20, 21].

The functional status was assessed with the FSS-ICU, which is composed of 5 tasks: rolling, supine to sit transfer, sit to stand transfer, sitting on the edge of bed and walking. Each functional task is rated using a scale ranging from 0 to 7, with 0 corresponding to unable to attempt or complete task due to weakness and 7 corresponding to complete independence. The total score varies from 0 to 35 points (completely independent) [22, 23]. This score presents high inter-rater reliability among physical therapists who routinely work in the ICU [24]. It is also an internally consistent, valid and responsive measure of physical function in critically ill patients. The minimum important difference ranges between 2.0 and 5.0 points [25].

The assessment of physical function was performed using the PFIT-s. This tool presents good inter-rater reliability [26] and consists of four-component outcome measure: sit-to-stand level of assistance (assistance); maximal marching on the spot duration and number of steps (cadence); shoulder flexion strength (shoulder); knee extension strength (knee), the strength is obtained based on the Oxford grading system [27–30]. Each component presents a score ranging from 0 to 3 (0 indicates the inability to perform the task or achievement with a maximum level of dependence and 3 indicates the task accomplishment without any difficulty). Its total score ranges from 0 to 12 points and can be converted to an interval scale of 0 and 10, where the minimum significant difference varies between 1.0 and 1.5 points [27–30]. The original 12-point version of the scale was used in this study.

### Linking process

At first the contents of the tools were identified and linked to the ICF. This process was conducted by two independent experienced evaluators in this methodology following the guidelines proposed by Cieza et al. [18]. The proposed method has two updates [17, 18] and is widely used to observe several instruments framework within the ICF [31–34]. The most recent version has ten rules for linking the information from the scale’s contents to the ICF. The main content definition for each tool item and its correspondence with ICF existing category are the major rules [18]. All the evaluators were instructed to independently link the instruments (MRC-ss, PFIT-s and FSS-ICU) to the ICF, while a third evaluator was available to be contacted in case of doubts and/or disagreements (supplement 1).

The ICF has two parts with two components in each: Part 1 – Functioning and Disability = body functions and structures, activities and participation; Part 2 – Contextual Factors = environmental factors, personal factors. Each component can be expressed in positive and negative terms and contains several domains. Each domain is made up of various categories that correspond to the classification units.

The ICF codes correspond to the category as a whole and the qualifiers are numeric codes that specify the extent or the magnitude of the problem in each category [10]. Qualifiers range from 0 to 4 as follows: 0 – no problem (0 to 4% impairment); 1 – mild problem (5 to 24% impairment); 2 – moderate problem (25–49% impairment); 3 – severe problem (50 to 95% impairment); and 4 – complete problem (96 to 100% impairment). Table 1 presents the established criteria for ICF qualifiers’ definition.

**Table 1:** Linking of the MRC-ss, FSS-ICU, and PFIT-s scales to ICF qualifiers

MEDICAL RESEARCH COUNCIL SUM SCORE (MRC-ss)		
*Score for all items*		*ICF Qualifier*
Grade 5		0
Grade 4		1
Grade 3		2
Grade 2		3
Grade 1 and 0		4
**FUNCTIONAL STATUS SCORE FOR THE ICU (FSS-ICU)**		
*Score for all items*		*ICF Qualifier*
7 = Complete independence 6 = Modified independence		0
5 = Supervision only 4 = Minimal assistance (patient performing ≥ 75% of work)		1
3 = Moderate assistance (patient performing 26 − 74% of work		2
2 = Maximal assistance (patient performing ≤ 25% of work)		3
1 = Complete dependence 0 = Unable to attempt or complete task due to weakness		4
**PHYSICAL FUNCTION IN ICU TEST-SCORED (PFIT-S)**		
*Item*	*Item score*	*ICF Qualifier*
1. Sit-to-stand assistance	0 = Unable to stand	4
	1 = Assistance of two people	3
	2 = Assistance of one person	Moderate assistance = 2 Minimal assistance = 1
	3 = No assistance required to stand	0
2. Marching on the spot cadence	0 = Unable to march on the spot	4
	1 = 0–49	3
	2 = 50 - <80	Between 50–60 = 2 Between 60–80 = 1
	3 = More than 80 steps/min	0
3. Shoulder flexion strength 4. Knee extension strength	0 = Grade 0, 1, or 2	Grade 0 and 1 = 4 Grade 2 = 3
	1 = Grade 3	2
	2 = Grade 4	1
	3 = Grade 5	0

### Data analysis

Each ICF category and qualifier was expressed in absolute and relative and frequencies. The scores of the tools were expressed as means ± standard deviation. The inter-rater agreement for the linking process was performed using the Kappa coefficient and interpreted as follows: values ≤ 0 as no agreement, 0.01–0.20 as none to slight, 0.21–0.40 as fair, 0.41– 0.60 as moderate, 0.61–0.80 as substantial, and 0.81–1.00 as almost perfect agreement. Reliability was estimated using the Rosner scale [35]. Data were analyzed using the Statistical Package for Social Sciences (SPSS) software, version 23.0 (IMB Corp, USA). For all analyzes, a significance level of p < 0.05 was considered.

## Results

### Linking of the instruments to the ICF

The ICF categories identified in the tools covered a total of 14 items. Common contents were identified in 13 of the 14 ICF categories (Table 2) and two were related to body functions, six to body structures and five to activities and participation.

**Table 2:** Contents of the MRC-ss, PFIT-s, and FSS-ICU instruments and ICF categories

ICF ITEMS	MRC-ss	PFIT-s	FSS-ICU
**BODY FUNCTIONS**			
b7300 - Muscle power functions	Peripheral muscle strength	Peripheral muscle strength	
b7601 - Control of complex voluntary movements		Marching on the spot cadence	
**BODY STRUCTURES**			
s7202 - Muscles of shoulder region	Shoulder abduction	Shoulder flexion	
s73002 - Muscles of upper arm	Elbow flexion		
s73012 - Muscles of upper forearm	Wrist extension		
s75002 - Muscles of thigh	Hip flexion		
s75012 - Muscles of lower leg	Knee extension	Knee extension	
s75022 - Muscles of ankle and foot	Ankle dorsiflexion		
**ACTIVITIES AND PARTICIPATION**			
d4103 - Sitting			Transfer from supine to sit
d4104 - Standing		Sit-to-stand transfer	Transfer from sit-to-stand transfer
d4107 - Rolling Over			Rolling
d4153 - Maintaining a sitting position			Sitting on the edge of the bed
d4500 - Walking			Ambulation

As shown in Table 2, the items of all instruments could be linked to ICF codes. However, some of the codes were suitable for more than one instrument. In these cases, only the instrument that best quantified the functional level was chosen as follows: the b7300 code was presented as the total score of the MRC-ss; the s75012 code was assessed according to the MRC-ss and only the result of the segment that obtained the highest result was used for data analysis; and the codes s7202 and d4104 were applied according to the FSS-ICU due to greater possibility of functional level specification.

As shown in Table 3, the inter-rater agreement was considered substantial for MRC-ss and PFIT-s linking to the ICF, and almost perfect for the FSS-ICU linking process.

**Table 3:** Common categories covered by the instruments and Kappa coefficients (k)

	MRC-ss (k = 0.665)	PFIT-s (k = 0.749)	FSS-ICU (k = 0.832)
**BODY FUNCTIONS**			
b7300 - Muscle power functions	**X**	**X**	
b7601 - Control of complex voluntary movements		**X**	
**BODY STRUCTURES**			
s7202 - Muscles of shoulder region	**X**	**X**	
s73002 - Muscles of upper arm	**X**		
s73012 - Muscles of upper forearm	**X**		
s75002 - Muscles of thigh	**X**		
s75012 - Muscles of lower leg	**X**	**X**	
s75022 - Muscles of ankle and foot	**X**		
**ACTIVITIES AND PARTICIPATION**			
d4103 - Sitting			**X**
d4104 - Standing		**X**	**X**
d4107 - Rolling over			**X**
d4153 - Maintaining a sitting position			**X**
d4500 - Walking			**X**

### Functional profile

Twenty-four in-patients (14 males) with mean age of 63.1 ± 8.9 years and hospital length of stay of 4.6 ± 2.7 days were included. The functional profile of the patients is shown in Table 4. The main causes of admission to the ICU were cardiac (62,5%), respiratory (25%) and postoperative conditions (12,5%). 100% of the sample presented comorbidities prior to hospitalization (diabetes mellitus – 100%, pneumonia – 20,8%, coronary artery disease – 62,5% and COPD – 8,3%).

**Table 4:** Functional profile of the sample according to ICF components and qualifiers

	0	1	2	3	4
**BODY FUNCTIONS**					
b7300 - Muscle power functions (MRC-ss)	2(8.3)	14(58.3)	5(20.8)	3(12.5)	0(0.0)
b7601 - Control of complex voluntary movements	3(12.5)	0(0.0)	10(41.6)	7(29.1)	4(16.6)
**BODY STRUCTURES**					
s7202 - Muscles of shoulder region (flexion)	4(16.6)	14(58.3)	5(20.8)	0(0.0)	1(4.1)
s7202 - Muscles of shoulder region (abduction)	5(20.8)	14(58.3)	5(20.8)	0(0.0)	0(0.0)
s73002 - Muscles of upper arm	11(45.8)	11(45.8)	2(8.3)	0(0.0)	0(0.0)
s73012 - Muscles of forearm	7(29.1)	14(58.3)	3(12.5)	0(0.0)	0(0.0)
s75002 - Muscles of thigh	5(20.8)	16(66.6)	3(12.5)	0(0.0)	0(0.0)
s75012 - Muscles of lower leg	12(50.0)	8(33.3)	3(12.5)	0(0.0)	1(4.1)
s75022 - Muscles of ankle and foot	10(41.6)	11(45.8)	3(12.5)	0(0.0)	0(0.0)
**ACTIVITIES AND PARTICIPATION** *					
d4103 - Sitting	18(75.0)	2(8.3)	3(12.5)	1(4.1)	0(0.0)
d4104 - Standing – (FSS)	16(66.6)	2(8.3)	3(12.5)	3(12.5)	0(0.0)
d4107 - Rolling Over	17(70.8)	3(12.5)	2(8.3)	2(8.3)	0(0.0)
d4153 - Maintaining a sitting position	21(87.5)	3(12.5)	0(0.0)	0(0.0)	0(0.0)
d4500 - Walking	18(75.0)	2(8.3)	1(4.1)	3(12.5)	0(0.0)

Data are shown as absolute (n) and relative frequencies (%). The qualifiers assigned to activities and participation refer to measures of functioning and disability (in CIF language)

As shown in Table 4, most of the patients (58.3%) presented mild problem regarding peripheral muscle strength, while moderate and severe problems were identified in 20.8% and 12.5%, respectively. These results agree with the classification of the MRC-ss instrument and are justified by the results of the body structures domain. For example, the ICF qualifiers 2 and 3 correspond to significant and severe weakness in the MRC-ss, respectively.

A mean score of 30.5 ± 7.4 was observed in the FSS-ICU instrument, indicating that the sample presented some degree of functional impairment. When measured using the ICF activities and participation domain, most of the sample (≥ 66.6%) was categorized as no problem (i.e., qualifier 0) for the following codes: d4103, d4104, d4107, d4153 and d4500. This was probably because the qualifier 0 was assigned to both the “total independence” and “modified independence” scores (i.e., use of supports, grids and aid devices during the task).

The results about physical functioning, according to PFIT-s, presented a mean score of 8.2 ± 2.4 (functional impairment) and none of the patients scored the maximum value. The functional impairment was also observed when assessing the items in the ICF model, especially in the b7601 code (stationary gait), since only three patients (12.5%) were categorized in the qualifier 0 (no problem) and the remaining patients in the qualifiers 2 to 4 (moderate to complete problem) (Table 4).

## Discussion

The ICF structure, created and recommended by the World Health Organization, defines functionality as a generic term for the interaction between three distinct constructs: body function, body structure and activities and participation [10].

The functional impairment due to critical illness leads to significant morbidity and burden for patients, caregivers and the society. As the number of ICU survivors is growing worldwide it becomes essential to standardize the functionality and physical function measurements [36]. Currently, there is not a single measurement tool available that can be used during the entire recovery journey. Therefore, it seems essential to consider the elements assessed under the ICF domains [36].

Electronic tools that aim to operationalize the ICF qualifiers in acute care settings can provide the functional profile and define the main objectives of the treatment interventions based on the ICF categories [37, 38]. These tools may contribute to reducing the time for filing the medical records, facilitate the registration of information in databases and share information between different medical sectors [38, 39]. However, the usefulness and applicability of this approach need to be explored. Therefore, studies classifying the patients’ health needs according to the ICF are essential to allow comparisons based on a universal language [40].

Clinicians and researchers can use appropriate ICF outcome measures to observe changes in the patients’ level of impairment, activity limitations and participation restrictions [8]. Parry et al. [36] identified several ICF domains in 11 of the most well-known physical function instruments and the following items corroborated with our results: d4103, d4153 and d4500 for FSS-ICU, as well as b7300 and d4103 for PFIT-s. In another study [41], data from 60 physical function instruments covered 26 ICF domains and 19 mobility subdomains. The b730, d4103, d4107, d4153, d4500 codes also corroborated with our study. However, the following items differed: b749, b455, d4, d4104, and d4508 for PFIT-s; d4, d4100, d4104 and d465 for FSS-ICU. As in the study conducted by Parry et al. [36], this review [41] did not mention the MRC-ss items.

Previous studies observed that FSS-ICU and PFIT-s present excellent validity for the ICU environment. These are promising functional measures and should be considered when measuring physical function in both clinical practice and research [42]. Nevertheless, the assessment of voluntary muscle strength at bedside using the MRC-ss [43, 44] predicts mortality, length of ICU and hospital stay and duration of mechanical ventilation [45].

The ICF categories used in our study corroborate with Paschoal et al., who identified the following relevant components and codes for acute and post-acute care in the Brazilian scenario [15]: body functions with codes b730 (muscle power functions) and b760 (control of voluntary movement functions); body structures with codes s720 (structure of shoulder region), s730 (structure of upper extremity) and s750 (structure of lower extremity); and activity and participation with codes d410 (changing basic body position), d415 (maintaining a body position) and d450 (walking). Despite this, it is important to note that it is mentioned in the recommendations that FCC-ICU and MRC-ss are used as tools in the neurological/neurosurgical and cardiovascular/cardiosurgical intensive care environments, respectively [41]. Only PFIT-s is not mentioned in these environments. Moreover, none of them were evaluated outside the ICU setting. Taking this for granted, our study is the pioneer in linking these instruments to the ICF and combining FSS-ICU, PFIT-s and MRC-ss in an assessment model based on the ICF.

Seguel et al. [9] conducted a study using FSS-ICU score with a methodology like ours, but the authors attributed the score 7 of the scale (complete independence) to the qualifier 0 (no problem) and the scores 4, 5, and 6 (from minimum assistance to modified independence) were assigned to the qualifier 1 (mild problem). In our study, the qualifier 0 (no problem) was composed of scores 7 and 6 (complete and modified independence) since the patient is considered independent in both scores, even though some walking or standing aid devices are used. Additionally, a 4% margin variation can be attributable to some possible events, such as imbalance or slow walking speed. On the other hand, both scores 4 and 5 were assigned as qualifier 1 in our study, indicating that the patient must perform at least 75% of the task alone. We believe this can be considered practicable since qualifier 1 allows a level of impairment up to 24%.

Some key considerations in choosing an instrument for application should be based on the purpose of the assessment, measurement properties, patient capability and clinical utility. Therefore, the use of MRC-ss on ICU admission and FSS-ICU and PFIT-s during hospitalization are recommended in clinical practice [36].

The MRC-ss is indicated by the most relevant studies for the clinical diagnosis of ICU-AW, as it is routinely used to screen for muscle weakness in critically ill patients [42, 43]. In addition to being a widely used method, it also has excellent inter-rater reliability for the overall score and is considered a sensitive method to assess the progression of rehabilitation for ICU-AW in patients who do not have enough strength to overcome gravity [20, 44].

FSS-ICU allows the assessment of physical function within 10 to 30 min of application (depending on the patient’s functional status) and is translated and validated for the Brazilian population [26]. It presents high inter-observer reliability among physical therapists working in ICU environments and excellent reliability for its use in critical care settings [24, 45]. In addition, this instrument has a low estimated ceiling and floor effect (below the acceptable cut-off point of 15%) at awakening and discharge from the ICU time points. This brings important value in evaluating the recovery process of patients and the effectiveness of a determined intervention. Floor or high ceiling effects indicate that the instrument is too challenging or too easy, respectively, limiting its ability to detect a change in patients’ physical function. Regarding FSS-ICU, it still presents evidence for a minimum important difference [8, 25, 36, 44, 45].

PFIT-s allows the assessment of the patient in 10 to 15 min, requiring only a stopwatch and a subjective exertion perception scale (optional). The ceiling and floor effects are also considered low (below the acceptable cut-off point of 15%) at awakening and discharge from the ICU time points, showing evidence of a minimal important difference. Furthermore, a study has shown that PFIT has convergent validity (moderate to high correlation with TUG, 6MWT and muscle strength by MRC-ss); divergent validity (low correlation with body mass index); high responsiveness over time (high effect size – 0.82) and predictive validity (association with peripheral muscle strength, hospital discharge, length of stay, and need for post-discharge rehabilitation) [29, 45].

Although this pilot study provides relevant contributions regarding the linking process of validated ICU instruments to the ICF, the categories used on our analysis were restricted to the body structures, body functions and activities and participation components without including the environmental factors. This was due to two main reasons: first, less approach is traditionally given to environmental factors in the hospital and intensive care setting; second, the tools investigated in our study concern particularly to functional tasks of bedridden patients. Even though health care professionals are aware of the potential impact of environmental factors on patients’ results and prognosis, the relevance of environmental issues in an ICU setting cannot be directly influenced by physical therapists since ICF provides broad definitions for the categories of the environmental factors component [39]. Therefore, future studies are needed to trace the functional profile of critically care patients using the environmental factors of ICF categories. Additionally, we suggest that future studies investigate the association between different categories and qualifiers and clinical outcomes.

## Conclusions

The proposed model combines the ICF with the contents of the most relevant tools used in critical care and ICU for physical function assessment. This study synthesizes and categorizes the most used instruments and presents a new proposal based on the ICF to guide future studies.

Although ICF tool is robust in describing the functionality in acute care settings, it is not widely used in this setting and is still not feasible in terms of environmental factors since the assessment is restricted to functions, body structures and activity and participation.

## Data Availability

The datasets used and/or analyzed during the current study are available by request from the corresponding author.

## Abbreviations

ICU: Intensive Care Units
ICF: International Classification of Functioning, Disability and Health
SIRS: Inflammatory Response Syndrome
MRC-ss: Medical Research Council sum score
FSS-ICU: Functional Status Score for the ICU
PFIT-s: Physical Function in ICU Test-scored
